# Understanding the Complexity of Teacher Emotions From Online Forums: A Computational Text Analysis Approach

**DOI:** 10.3389/fpsyg.2020.00921

**Published:** 2020-06-05

**Authors:** Zixi Chen, Xiaolin Shi, Wenwen Zhang, Liaojian Qu

**Affiliations:** ^1^Department of Counseling, Educational Psychology, and Special Education, Michigan State University, Lansing, MI, United States; ^2^School of Hospitality and Tourism Management, College of Health and Human Sciences, Purdue University, West Lafayette, IN, United States; ^3^Department of Public Affairs Administration, South China Agriculture University, Guangzhou, China; ^4^Department of Education, Jiangnan University, Wuxi, China

**Keywords:** emotion multi-dimensionality, emotion dynamics, emotion-rich big data, computational text analysis, teacher out-of-school emotions

## Abstract

Teacher emotions are complex as emotions are unique to individuals, situated within specific contexts, and vary over time. This study contributed in synthesizing theories of the complexity in two characteristics of multi-dimensionality and dynamics. Further, we provided large-scale empirical evidence by employing big data and computational text analysis. The data contained around one million teachers’ online posts from 2007 to 2018. It was scraped from three representative forums of teachers’ workplace events and personal life occasions in a popular American teacher website. By conducting thread-level sentiment analysis in forums, we computed word-frequency-based eight discrete emotions ratios (i.e., anger, anticipation, disgust, fear, joy, sadness, surprise, and trust) and the degrees of sentiment polarity (i.e., positive, negative, and neutral). We then used latent Dirichlet allocation for topic classifications. These topics, proxies of contexts, covered a holistic range of teachers’ real-life events. Some topics are in the main interest of scholars, such as teachers’ professional development and students’ behavioral management. This paper is also the first to include the less scholarly studied contexts like professional dressing advice and holiday choices. Then, we examined and visualized variations of emotions and sentiments across 30 topics along with three scales of time (i.e., calendar year, calendar month, and academic semesters). The results showed that teachers tended to have positive sentiments in the online professional community across the past decade, but all eight discrete emotions were presented. The compositions of the specific emotion types varied across topics and time. Regarding the topics of students’ behavior issues, teachers’ negative emotions’ ratios were higher compared when it was presented in other topics. Their negative emotions also peaked during semesters. The forum of teachers’ personal lives had positive emotions pronounced across topics and peaked during the wintertime. This paper summarized the evidenced multi-dimensionality characteristic with the multiple types of emotions as compositions and varying degrees of sentiment polarity of teachers. The dynamics characteristic is that teachers’ emotions vary across contexts from their workplace to their personal lives and over time. These two characteristics of complexity also suggested potential interplay effects among emotions and across contexts over time.

## Introduction

The studies on teachers’ emotions have grown increasingly over the past decades. Accumulated scientific evidence showed the critical roles that teachers’ emotions play in many aspects of their professional life, including teaching training (Darling-Hammond, 2001), teaching satisfaction (Yin et al., 2013; Yin, 2015), school and policy climate (Lee and Yin, 2011; Rawolle, 2013), and turnover (Richardson et al., 2008). Besides the influences on teachers’ professional life, teachers’ emotions also related to students’ emotions (Frenzel et al., 2009), students’ learning (Pekrun et al., 2011), and the interrelationship between teachers and students (Yan et al., 2011).

Together with the expanding research on the many manifestations of teachers’ emotions within educational contexts, scholars have forwarded a critical viewpoint of the complexity of teacher emotions (Sutton and Wheatley, 2003; Schutz and Zembylas, 2009; Fried et al., 2015), that is: teachers experience varying emotions within specific contexts of particular times. Fried et al. (2015) summarized that teachers’ emotions are complex as emotions can evolve over time, are a unique phenomenon for each individual, are contextualized, and are multi-componential (e.g., Sutton and Wheatley, 2003; Frenzel et al., 2015; Chen, 2016). Several studies have examined multiple categories of teachers’ emotions in general teaching contexts (Chen, 2016; Frenzel et al., 2016) and raised the importance of studying teachers’ emotions across time (Scott and Sutton, 2009). Scholars also called for future studies on the complexity characteristics with building comprehensive conceptual frameworks and using real-life contexts, multi-sourced methods, reliable measures, and qualified datasets (Zembylas, 2003; Frenzel, 2014; Pekrun and Schutz, 2007).

Meanwhile, the emergence of big data provides unprecedented opportunities for social science scholars to study people’s behaviors, emotions, opinions, cognitions, interactions, and experiences (Chen and Wojcik, 2016; Mahmoodi et al., 2017; Salganik, 2019). Big data analytics in social science fields developed computational social science tools to integrate machine learning with social science traditional inquires (Lazer et al., 2009; Cheung and Jak, 2019). For example, computational text analysis, such as topic modeling and sentiment analysis, was increasingly used for detecting semantic topics and sentiments in unstructured text data within the social science paradigm (Pennebaker et al., 2003; Burnap et al., 2015; Chen and Wojcik, 2016; Algaba et al., 2019).

The contributions of the current paper to the studies of the complexity of teacher emotions are twofold. First, we provided a holistic picture of the complex characteristics of teachers’ emotions. Building on the Affective Events Theory (AET) of Weiss and Cropanzano (1996) and the appraisal model of Frenzel (2014), this paper integrated AET and the appraisal model to propose that the complexity of teachers’ emotions can be elaborated into two features: multi-dimensionality and dynamics (Sutton and Wheatley, 2003; Fried et al., 2015). Rather than viewing teachers’ emotions by examining a single emotion, we shift to analyze teachers’ multiple emotions simultaneously under three settings, including teachers’ teaching in class, professional development, and personal life. Second, this paper provided an example of the affordances of big data and computational text analysis tools in teachers’ emotions studies. The big data were scraped from three public online forums of teachers. Although big data analysis is normally considered as data-driven (Lazer et al., 2009), this paper adopted a combination of inductive and deductive approaches to underpin the current theories and uncover new findings.

This study aims to use big data analytics, including sentiment analysis and topic modeling, to explore the complexity of teachers’ emotions. To fully depict this complexity and its two features of multi-dimensionality and dynamics, this article proposes three research questions:

1. What are the compositions of emotions and sentiment polarity in teachers’ online forums?
2. How do these compositions and polarities differ across workplace contexts in schools and personal life events?
3. How do these compositions and polarities differ across time?

## The Complexity of Teacher Emotions

### The Multi-Dimensionality of Teacher Emotions

Weiss and Cropanzano (1996) and Fredrickson (2001) stated that emotions are affective states and a subset of the affective phenomenon. Specifically, Fredrickson (2001) argued that emotion is a construct with multiple types, and its formation starts from an individual’s assessment of an event’s meaning and potential influence. This was called an appraisal process, which can be conscious or unconscious, triggering affective reactions through facial expressions, cognitive behaviors, or physiological changes (Fredrickson, 2001). Frenzel (2014) proposed five appraisal dimensions that are closely related to teachers’ instructional behavior, such as classroom management and motivational stimulation. The five appraisal dimensions are goal consistency, goal conduciveness, coping potential, goal attainment, and goal importance (Frenzel, 2014). Depending on these appraisals, teachers display different emotional responses, which is consistent with Weiss and Cropanzano’s (1996) AET. AET theorized that due to the appraisal of different affective events, individuals experience varying types of emotions simultaneously. For example, a teacher may experience joy when students’ learning results are consistent with the teaching goals (i.e., the goal consistency appraisal) (Frenzel, 2014). However, when the goals between teachers and students are not consistent, teachers may experience negative emotions such as anger and anxiety. Additionally, teachers may not express the same emotions for the same event because of their different appraisal processes (Sutton and Wheatley, 2003).

Moreover, the multi-dimensionality of teachers’ emotions indicates that emotions may interact with each other. One way is that a type of emotion may eliminate or cancel out the effect of another type of emotion and finally influence the impacts of emotions. For example, a teacher who worries about the low payment and thinks about changing jobs may be compensated by the satisfaction of his/her students’ achievement and thus decides to stay. This phenomenon can be explained by using the broaden-and-build theory developed by Fredrickson and Joiner (2002). This theory stated that positive emotions broaden individuals’ attention and creative thinking, which facilitates the ability to deal with negative emotions (Fredrickson and Joiner, 2002). The other way is that a type of emotion may enhance or magnify the effects of other types of emotions (Fredrickson and Joiner, 2002). If only a single type of emotion or sentiment valence is evidenced, we would not necessarily know about the existence of other types of emotions.

Many empirical studies of teachers’ emotions reflected this multi-dimensionality characteristic of emotions by measuring discrete types of emotions within specific educational contexts (e.g., Brooks et al., 2008; Lee and Yin, 2011; Yan et al., 2011). For example, Frenzel et al. (2016) created a teacher emotion scale using enjoyment, anger, and anxiety. Chen (2016) argued that teachers’ emotions had five dimensions: joy, love, sadness, anger, and fear. Situated within specific contexts, Brooks et al. (2008) studied how an education reform influences teachers’ sense of alienation and fear. Hagenauer and Volet (2014) found that, among university teachers, joy, happiness, and hope are the most frequently mentioned positive emotions, while annoyance and insecurity are the most frequently mentioned negative emotions. Instead of discrete emotions, some earlier studies examined a dichotomous scale of positive and negative called polarity or valence (Frenzel, 2014).

### The Dynamics of Teacher Emotions

The previous studies provided essential insights into the multidimensional nature of teachers’ emotions. Other studies called attention to the emotions’ changing nature and the constitution of emotions across persons and situations. Frenzel et al. (2015) argued that teachers’ emotions are a function of both the person and the situation, which means that teachers’ emotions may vary based on many situational factors, such as the subject of teaching and the student group they are teaching. Building on the multidimensional nature of teachers’ emotions and responding to the call for future research to use a multidimensional perspective to study teachers’ emotions (Sutton and Wheatley, 2003), this paper states in this section that teachers express multiple types of emotions within or across different work events and time.

#### Emotions Across Work and Personal Life Contexts

Based on the AET, the appraisal process also emphasizes the relationship between work events and emotions. Notably, the AET states that the primary appraisal is influenced by the relevance of the work events to individuals’ overall well-being and, later, the secondary appraisal helps individuals to determine the types of emotions they will experience. For example, students’ learning performance can trigger teachers’ primary appraisal process because it can be viewed as a work event that is relevant to teachers’ emotions. Both Chen (2016) and Frenzel et al. (2015) stated that teachers’ emotions are embedded within the environment and vary based on the work context. Therefore, teachers may either perceive the work events as positive or negative, depending on the influence of the work contexts. The process leads to the happening of the secondary appraisal process, which determines the types of emotions that teachers will display. Cross and Hong (2012) also commented that the appraisal process highlights the critical role of the context or environment in influencing teachers’ emotions. One reflection of the teachers’ appraisal process is that, when the work event changes from one to another, the appraisal process of teachers change. In turn, the corresponding emotions of teachers change. With a person–environment fit argument, Frenzel et al. (2015) found that teachers’ emotions, such as joy and anger, varied within individuals and were influenced by specific attributes and status of the educational contexts, including the characteristics of students and the subject of the class that they teach.

It has been long recognized that teachers’ work performances, identity, perceptions, and practices in schools are largely integrated with their personal lives and experiences (Clandinin, 1985; Pajak and Blase, 1989; Connelly et al., 1997; Day et al., 2006; Tour, 2015). In other words, teachers’ personal lives and their professional activities and experiences are integrated. Hence, teachers’ emotions situated from their family events may also be relevant to understand teachers’ emotional manifestos within their professional activities. However, teachers’ emotions in their personal lives were less evidenced in current literature. This study attempted to show teachers’ emotional compositions within personal life events and further compare these with the ones within workplace activities in school context. The second research question served this goal.

#### Emotions Across Time

Emotions may evolve as time changes, and the context or related environmental factors change. This is consistent with Meyer and Turner’s (2006) theoretical argument that teacher emotions are dynamic and evolve over time. Specifically, they argued that students’ motivation to learn, teachers’ motivation to teach, and how well the two parties communicate with each other determine the climate of learning. Therefore, teacher emotions may vary across time, depending on whether the climate of learning is viewed as positive or negative. Frenzel et al. (2015) found that as the work contexts change, such as new assignments of students, teachers’ emotions vary across semesters or academic years instead of being static. Teacher emotions are not a stable phenomenon on a daily basis. Schmidt et al. (2017) found that the day-to-day experience of early career teachers’ emotional uplifts and hassles significantly predicted their daily emotional exhaustion. Simbula (2010) also found that teachers’ emotional exhaustion fluctuates daily.

Although the aforementioned research has provided a micro-level evidence in teachers’ emotional variations within mainly daily-based professional tasks, we still need more knowledge on the dynamics of teacher emotions across a more extended time range. The teaching profession has particular work events in certain months or semesters in the academic year cycle, such as students testing each academic year which may happen in April for state-level evaluation and at the end of spring and fall semesters as final tests. These seasonal work events were found to affect teachers’ emotions. During student testing and teacher evaluation, teachers experienced anxiety and pressure, which led to emotional exhaustion and, finally, turnover behaviors (Skaalvik and Skaalvik, 2011; Wronowski and Urick, 2019). Diary studies that commonly collect data for 14 consecutive days are less likely to apply to a general teaching semester and have possible long-term effects.

It is not uncommon that current teacher emotions literature employed data collected from a few short time points and particular events (Sutton and Wheatley, 2003; Frenzel, 2014; Fried et al., 2015). This convention leads to scholarly evidence in single types of emotions or polarity within limited contexts. There was less empirical evidence in long-term teacher emotional depictions and its impact on teachers’ professions and lives. Therefore, traditional data collection approaches, such as interviews and surveys, are not efficient and sufficient in providing evidence of the complexity of teacher emotions. Conventional data are further limited in generalizability, either for making inferences to other groups of teachers or for applying it into other educational contexts. Regarding time scales, these require longitudinal datasets that cover the complete cyclical professional activities of teachers, such as academic semesters across multiple years. If teachers’ emotions are only presented as incomplete, our understanding of its antecedents and influences will be biased. Most importantly, teachers and students who are in need may not be offered adequate help.

**FIGURE 1:**
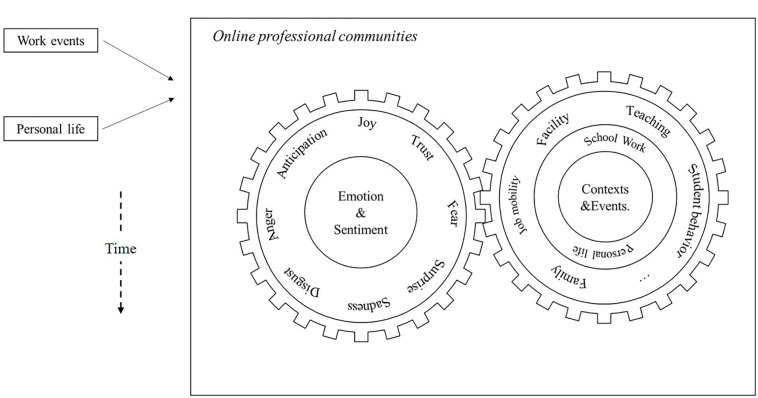
Conceptual diagram of multidimensionality and dynamics of teacher emotions.

In summary, the theoretical framework is built upon AET (Weiss and Cropanzano, 1996) and the appraisal model of Frenzel (2014). The integration of these two theories indicated that teachers could experience multiple emotions simultaneously (i.e., multi-dimensionality). Moreover, teachers’ emotions can vary across work context and time (i.e., dynamics). We demonstrate this conceptual model of the complexity of teacher emotions in Figure 1. As shown in the diagram, the left wheel represents the multi-dimensionality of teachers’ emotions through multiple types of emotions and polarity of sentiments. This diagram further represents the dynamics of emotions through the right wheel and the time arrows on the lower left. Specifically, the right wheel contains the varying contexts of teachers’ emotions embedded within. When a context and/or the time change, the left emotion wheel moves as the compositions of emotions change.

## Emotion-Rich Big Data

In online space, such as social media and teachers’ blogs, teachers bonded with trust and emotional support along with their interactions, knowledge sharing, and resource acquisition (Booth, 2012; Macià and García, 2016; Hu et al., 2018). Particularly, Hur and Brush (2009) found that one of the top reasons, but less studied, of teacher participation in online communities was sharing emotions related to teaching activities. Davis (2015) highlighted that teachers’ online communities created a sense of belonging as emotional support among teachers. Teachers’ activities online left digital prints in various forms, including text, pictures, and audio. Moreover, big data consist of emotion-rich messages and information that cover a wide range of topics and timestream benefit for the community of psychological research (Chen and Wojcik, 2016; Adjerid and Kelley, 2018; Hu et al., 2018).

We here extracted several sentences from an anonymous user’s public post from our sample data as a prelude. It demonstrates the emotion-rich information that teachers’ online communities can carry. This post said: “As an introvert, this really affects my mood and health. I’m scared of ending up in the hospital. I decided to look for other teaching jobs.” In this example, the data provided information of emotions, contexts of time and events, and their impacts on teachers’ decisions.

The rich emotion and event measures from online platforms have shown advantages in reliability, validity, and efficiency. Hur and Brush (2009) also noted that since the online communities are anonymous, teachers are more likely to freely share emotions than in the ones using their real names. It is a less transparent benefit of using big data for social and behavioral research that people are more likely to show authentic information than if they were surveyed or interviewed. Therefore, big data pertaining to teachers’ at-the-moment real-life occasions can uncover new or hidden qualified features of emotion constructs than the traditionally acquired data.

Although big data have much potential to help the current need for teacher emotions’ studies, no teacher emotions big data analytic work has been conducted as of now to the authors’ knowledge. This could be due to the many challenges of using big data in the social sciences. One challenge could be the requirement of skills in big data acquisition and analytics. As detailed in later sections, this paper scraped public online data and organized it into a structured longitudinal dataset. Then, we utilized computational text analysis, including sentiment analysis and topic modeling, to compute emotion estimates and further analyzed the results with traditional quantitative methods.

The other challenge calls for the development of methodological frameworks of integrating social science research and big data analytics (Mahmoodi et al., 2017; Nelson, 2017). It relates to the data-driven and theory-driven debate in using big data analytics in social science research. Most of the time, big data analytics are data-driven as the traditions in machine learning, bottom-up, and inductive (Lazer et al., 2009; Moessner et al., 2018; Rodriguez and Storer, 2020). In contrast, the traditional social and behavioral sciences, including psychology, are most often theory- or hypothesis-driven, top-down, and deductive (Chen and Wojcik, 2016; Mahmoodi et al., 2017). Instead of staying on these different traditions, recent discussions extend to the integration of these traditions (Salganik, 2019; Rodriguez and Storer, 2020). Research in psychology using big data can initiate research designs based on established theories and hypotheses to provide further evidence or even theoretical and measurement development (Adjerid and Kelley, 2018). The current paper adopted this approach: a theory-driven initiative underpinned and built by data-driven evidence.

Aside from the many benefits of big data, the veracity or quality of big data is worth discussing as the data were not initially designed and generated based on research (Chen and Wojcik, 2016). Additionally, big data could be unstructured, thus needing to be cleaned and restructured for the particular analysis purposes (Taleb et al., 2015; Chen and Wojcik, 2016). The current paper paid much attention to data quality issues in raw data cleaning and text data processing as discussed in the following sections. The contemporary data science field has developed many statistical approaches for modeling selection, while the current paper further discussed the reliability and the validity of big data measurements and modeling with strong foci on meaningful social interpretations that build upon the computational grounded theory in Nelson (2017).

## Data and Methods

This paper utilized online data from a popular American-based teacher website. We then conducted the sentiment analysis to compute emotions’ compositions as ratios and sentiment polarity. The sentiment polarity had two measures: estimated polarity scores (i.e., degrees of positive and negative) of threads and percentages of positive, negative, and neutral threads. Further, to answer the second question of dynamics across contexts, we firstly employed latent Dirichlet allocation (LDA) to reduce the complex forum text data into interpretable topics. These topics were treated as proxies of contexts that each thread was embedded within. In terms of emotions’ dynamic in time, we developed three scales: calendar years, calendar month, and academic semesters. The academic semesters were defined into fall semesters (September to November), spring semesters (January to May), summer breaks (June to August), and winter breaks (December). Finally, variations of teachers’ emotions across contexts and time were examined through ANOVA tests and presented in visualizations (i.e., radar charts, time trending plots, and boxplots).

### Big Data Acquisition, Cleaning, and Organization

Used Python language, we scraped 66,515 threads from the three public forums. Two of the forums are workplace events: general teaching activities in classrooms and teachers’ professional development. The school contexts and events in these two forums could cover the most-studied professional events of teachers in teachers’ emotional studies, as discussed in the above contemporary literature. The third forum is teachers’ out-of-school life, in which teachers examine their personal life events, such as holidays and family, and personal health concerns. This forum provides a novel perspective to learn teachers’ emotions outside of school buildings, which could have different emotional manifestos than the ones from professional events.

**FIGURE 2:**
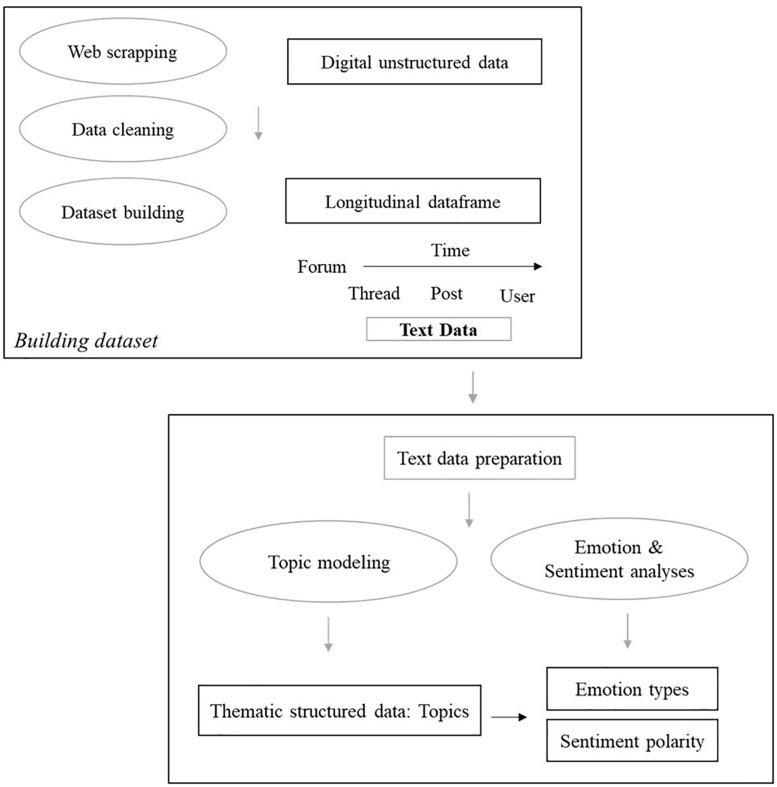
Organizing Unstructured Big data into structured Longitudinal dataset for computational text Analyses.

As shown in the top panel of Figure 2, the scraped digital unstructured data were organized into a structured longitudinal format. The data cleaning process followed the big data quality dimensions summarized in Taleb et al. (2015), including measurement accuracy, timeliness, consistency, and completeness. The accuracy of measures involved text cleaning of gibberish symbols or letters and removing of website advertisements. The timeliness dimension required that the measures are time windows that are up to date or relevant to research questions. The sample data satisfied this dimension as these contained all threads of each forum from when the first thread was created to the last day of 2018 when the research question of emotion dynamics was answered. The data consistency and completeness dimensions are closely related to the structural building of the scraped data as described below.

The three forums were considered independent of each other since they are pre-determined with unique themes. Within forums, teachers generate interactive discourses through posts around a common subject within a thread. In the cleaned dataset, each thread and the posts within each thread were assigned with unique thread- and post-level identification numbers by the website. Besides the identification numbers, each thread and the posts within it have corresponding variables of users and created time. Notably, the discourses within a thread were not necessarily formed in a star structure where all the following posts directed to the starting post. The following posts can point to each other. Thus, the structure of a thread is interactive instead of being directed in one way. This study thus further argued that the posts within threads are dependent as they share common interests, while threads are independent if they hold individual interests. Therefore, we aggregated posts to thread level for later analysis. In the bottom panel of Figure 2, the structured longitudinal text data were then used for text analysis to compute sentiment estimates and topics.

Table 1 presents the descriptive statistics of the three forums. Forum 1 of general teaching in classrooms is the most popular one, which has the largest total number of threads and the highest average number of threads per month and year. Forum 2 is the most prevalent as it has the largest number of unique participants and also a comparable large number of threads. This forum has professional development-relevant threads. Forum 3 is for discussions on teachers’ personal lives. While it has the lowest number of users, those users are active as they, on average, created around three times more posts than those of the other two forums’ users. The lengths of the threads in the three forums are relatively similar in terms of the average word count per thread.

**TABLE 1:** Descriptive statistics of the sample big data.

	Forum 1	Forum 2	Forum 3
	
	Teaching in class	Professional development	Personal life
Threads time range (year)	2007–2018	2007–2018	2007–2016
Total posts	342,248	226,928	291,111
Total threads	26,471	23,187	16,857
Total unique users	7,447	8,830	2,108
Average number of threads per month	2,228	1,932	1,405
Average number of threads per year	2,228	1,927	1,686
Average word count per thread	1,103	898	1,053
Average post number per thread	13	10	17
Average user number per thread	4	3	8
Average post number per user	46	26	138

Additionally, the peak of thread numbers happened during 2008–2012 at around 4,000 threads that were generated every year in all three forums. The number of threads per year then continuously declined in the following years. Within a year, teachers were most active during the summertime of June and July and the least in December.

### Sentiment Analysis: Emotion Compositions and Sentiment Polarity

Psychology and linguistic researchers have examined the emotional information carried in natural language with many computer-aided text analysis tools a long time ago (Pennebaker et al., 2003). In this study, we utilized a lexicon-based sentiment analysis approach that can obtain the sentiment polarity and emotion types of texts through the words within it (Sadia et al., 2018). In the analysis, a word associated with any sentiments or emotions is called a polarized term; otherwise, it is a neutral term. However, a single word itself is not sufficient to capture the true sentiment or emotions of a sentence, a paragraph, or a passage (Zhu et al., 2014). If a teacher wrote “I don’t like teaching math,” the negative term of “not” would invert the sentiment meaning of “like” from positive to negative. It is a negation situation: “not” and other similar words like “never,” “nor,” and “no” are negator terms. Other words also influence the degree of sentiments and emotions, such as valence shifters and adversative conjunctions. Unlike conventional text processing, where negator words are commonly deleted as a part of stop words, this paper kept the negator words. We also replaced words like “couldn’t” into “could not” to keep “not” for negative expressions. Negations through hyphens, like “non-,” are also considered.

**FIGURE 3:**
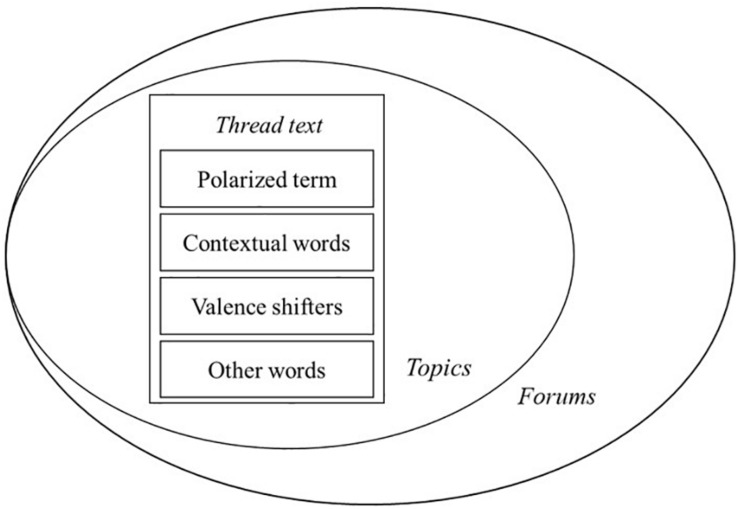
Contextual information of thread.

Additionally, we considered valence shifters and adversative conjunctions. Valence shifters like “slightly” and “very” influence the degree of sentiment. Adversative conjunctions, such as “but” and “however,” may contrast a statement. Therefore, instead of matching single words, this paper took into account the surrounding words as contextual information to improve the identification and the estimation of sentiments and emotions. The contextual words techniques in the sentiment analysis aligned with the study of Pennebaker et al. (2003), where they extracted emotional, social identity, and cognitive style features from people’s particles–parts of speech. Figure 3 illustrates the three layers of contextual information a thread had: contextual words, topics, and forums. They together aided in defining the contexts of teachers’ emotions.

Conducted through an R package called “sentimentr” (Rinker, 2017), we specified the analyses for each thread with counting a range of 13 contextual words around any single word within individual sentences. The analyses produce counts of words, counts of words in each emotion type, and sentiment polarity scores. Additionally, as compared to that of Naldi (2019), the package “sentimentr” is the only package to properly account for negators among four commonly used R packages for sentiment analysis.

The chosen lexicon for tagging polarized terms is called NRC. It was initiated from crowdsourcing on Mechanical Turk and annotated about 25,000 English words for emotional labels (Plutchik, 1962, 2001; Mohammad and Turney, 2010, 2013). Each word is labeled with a sentiment polarity of positive or negative and also the eight discrete emotion types from the emotion categories of Plutchik (1994), including fear, anger, disgust, sadness, acceptance, anticipation, joy, and surprise. These emotion categories have been used in some teachers’ emotions studies, such as those of Gross and Lo (2018) and Ihtiyaroglu (2019). A word may have one polarity or emotion type or multiple ones. For example, the word “abandon” is labeled with a negative polarity and classified into emotions of fear and sadness. The word “abundance” has polarity labels of both positive and negative. The corresponding emotion types are also multiple: anticipation, joy, trust, and disgust. In this lexicon, there are more negative affection-associated words than the positive ones. The unbalance of sentiment polarities in the English language was considered in the analyses by using proportion inverse weighting.

### Topic Modeling: Workplace and Personal Life Contexts

Topic modeling, such as latent Dirichlet allocation, is commonly used with machine learning or deep learning algorithms to calculate the latent structure (i.e., patterns or topics) of text (Blei et al., 2003). In proceeding to analyze the compositions of teachers’ emotions within specific contexts for the argument of dynamics, we employed LDA for the topic classifications of the threads. The basic idea of LDA is that words that belong to a given topic are more likely to appear in the same document than other words from other documents. With iterative computing, LDA produces the probability distributions of documents for multiple topics and the probability distributions of words that compose those topics. Compared with manual taxonomy, LDA is superior in revealing a variety of perspectives from large volumes of text data in an efficient, economical, and less subjectively biased way (Iliev et al., 2015; Nelson, 2017). Additionally, it helps disclose any novel topics that are less studied.

Text processing in LDA includes lemmatization and the removal of website links, stopwords, and punctuations. Lemmatization shortens each word into its basic forms. For example, “testing” and “test” will be reduced into a common form of “test.” Stopwords such as “a/an” and “you” are words that generally do not carry content information. Finally, observing from the frequency distribution of words, we truncated the top 10% most common words and the top 5% least common ones to improve topic classification (Madsen et al., 2004; Song, 2008).

In applications of topic modeling for social science studies, it is critical to make judgments of setting the number of topics and interpret arbitrary topics with meanings for qualified model selections (Grimmer and King, 2011; Grimmer and Stewart, 2013; DiMaggio, 2015; Baumer et al., 2017; Nelson, 2017). Nelson (2017) developed a three-step methodological process, named as computational grounded theory, to develop and interpret meaningful and reliable computer-aided classified patterns in social science studies. The first step is inductive and exploratory, where preliminary content patterns are produced from the algorithmic-based machine learning methods. Then, researchers turn to a deep reading of the outputs to refine the identified patterns and provide plausible interpretations. The third step is inductive. Researchers may use supervised machine learning with human-coded data to confirm that the identified and the interpreted patterns are reliable. The current study also weighs heavily on meaningful interpretation capacity instead of purely judging based on arbitrary modeling of fit values.

Therefore, along with the steps defined in the computational grounded theory framework, we further chose two quality measures of model selection for defining an appropriate topic number and sociologically meaningful topics: the degree of associations of the top words within each topic (i.e., consistency or cohesive) and how distinguishable those top words are for each topic (i.e., differentiation or exclusive) (Gerring, 2001; Roberts et al., 2014). For example, a reasonable topic should have words that are highly related and relevant to substantial interpretations. Also, if two topics have many overlapping most relevant words, then these two topics are less efficient as they are non-distinguishable. Therefore, the ideal model should produce a reasonable number of topics that are meaningful and distinguishable. This paper combined the above guidance to secure the reliability of the topic modeling. The model selection and the evaluation process are described below.

With prior knowledge of each forum through in-depth reading, we firstly conducted three LDAs for each forum with reasonable topic numbers of 10, 20, and 30. Then, we listed down the top 25 most frequent words with the highest probability in each topic to gain primary judgments of the topics’ meaning and to what degree a topic can be distinguished from other topics. Meanwhile, we randomly selected 20 threads from the 30 threads which have the highest probabilities belonging to a topic. Two authors then deeply read and discussed the original content of each thread for meaningful interpretations. The other 10 threads in each topic were used as validation evidence for topic interpretations. Lastly, we then found that the models with the topic number of 10 had the most interpretable and efficient results across the three forums. Therefore, this paper used the LDA outputs with 10 topics as the proxies of the workplace or personal life contexts. The 30 topics with interpretations and their prevalence percentages within forums are demonstrated in Supplementary Appendix A.

## Results

### Emotions in Teacher Online Forums

The emotion analysis computed the ratios of eight discrete emotions. To present the amount of these emotion types without the bias of language, we further weighted the percentages of the emotion types by the percentages of emotion types from the lexicon NRC. For example, anger’s weighted ratio was calculated through dividing the anger emotion percentage in a forum by the anger percentage in NRC. Table 2 shows the overall pairwise correlations of emotions based on the weighted ratio values from all threads of the three forums. The negative emotions, including anger, sadness, disgust, and fear, were strongly and positively correlated with each other. Similarly, we grouped anticipation, joy, trust, and surprise as positive emotions as they were strongly and positively correlated (*p* < 0.05). Their correlations with negative emotions are generally significant but close to zero. Similar patterns applied to the correlation matrices of each forum, although slight variations exist.

**TABLE 2:** Pairwise correlation matrix of weighted ratios of emotions.

	Anger	Sadness	Disgust	Fear	Surprise	Anticipation	Joy	Trust
Anger	1							
Sadness	0.44*	1						
Disgust	0.47*	0.55*	1					
Fear	0.68*	0.49*	0.42*	1				
Surprise	0.04*	0.04*	0.04*	0.04*	1			
Anticipation	< −0.01	< −0.01*	< −0.01*	0.06*	0.72*	1		
Joy	<0.01	<0.01	<0.01	0.01*	0.77*	0.76*	1	
Trust	−0.05*	−0.07*	−0.06*	−0.04*	0.47*	0.50*	0.56*	1

*p < 0.05.

In Table 3, we show the descriptive statistics of the compositions of emotions and sentiment polarity at the forum level. Based on the frequencies of the tagged emotional words through NRC, each forum contains around 10% of emotional-related information. Although forum 3 on teachers’ personal life has the smallest number of words in the threads, the percentage of emotion words is the highest. It seems like teachers are more likely to produce emotion-relevant information when talking about their non-teaching lives. We further rescaled the weighted ratios of emotions into percentages within each forum and presented them in parentheses. Then, the compositions of emotions were thus allowed to be compared within and across forums. All three forums have much fewer negative emotions than positive ones. The weighted ratios and percentages of each positive emotion were generally four to five times larger than all the negative ones. Accumulative percentages revealed the same pattern. Additionally, compared with the lexicon NRC, the percentages of negative emotions in the teachers’ sample text data are around five times less. Among the emotions, joy was the most prevalent one, and disgust is the least. Forum 2 has the least ratios or percentages of negative emotions. In forum 3, teachers tend to express more negative emotions about their personal lives than in the other two forums, where the latter ones are related to professional activities.

**TABLE 3:** Descriptive statistics of emotion compositions and sentiment polarity in forums.

	Forum 1	Forum 2	Forum 3
	
	Teaching in class	Professional development	Personal life
Emotion words percentage (%)	10.39	10.62	11.49
**Emotion types (NRC%)**	**Weighted ratio of emotion types (%)**		
Anger (27.94)	2.34 (4.72)	1.74 (3.34)	2.87 (5.09)
Sadness (26.69)	3.20 (6.45)	2.64 (5.05)	3.87 (6.86)
Disgust (23.71)	1.81 (3.66)	1.41 (2.70)	2.56 (4.53)
Fear (33.07)	2.50 (5.04)	1.98 (3.80)	3.10 (5.50)
Accumulative ratio (%)	9.85 (19.87)	7.77 (14.89)	12.4 (21.98)
Surprise (11.97)	7.37 (14.87)	8.81 (16.89)	8.82 (15.63)
Anticipation (18.80)	10.58 (21.34)	12.14 (23.26)	12.12 (21.47)
Joy (15.44)	11.46 (23.13)	12.23 (23.44)	13.88 (24.59)
Trust (27.58)	10.31 (20.80)	11.23 (21.52)	9.22 (16.34)
Accumulative ratio (%)	49.57 (100)	52.19 (100)	56.44 (100)
**SENTIMENT POLARITY STATISTICS**			
Mean	0.20	0.25	0.16
Standard deviation	0.31	0.33	0.30
Positive percentage	91.52	94.95	85.40
Negative percentage	7.34	4.26	13.55
Neutral percentage	1.14	0.79	1.05
Range	(−0.80, 1.22)	(−1.00, 1.31)	(−0.81, 1.26)

Consistently, all three forums have a positive average polarity, which implied that the overall sentiment of teachers’ online discourses tends to be positive. While variations exist across forums, forum 3 has the lowest positive sentiment polarity, and forum 2 has the highest one. At the forum level, positive polarity was dominant, while negative and neutral ones together accounted for only around 10% or less.

### Emotions Across Contexts

The weighted ratios of positive emotions were higher than the negative ones in all contexts (i.e., topics). Supplementary Appendix A contains the weighted ratio of emotions of all 30 topics. However, there were noticeable cross-context variations in the compositions of emotions and sentiment polarity scores. Two-way ANOVA tests were used to statistically examine the sentiment polarity variations across topics and time scales. For all the three forums, the sentiment polarity scores were significantly different across topics (*p* < 0.0001).

#### Emotions Within School Contexts

**FIGURE 4:**
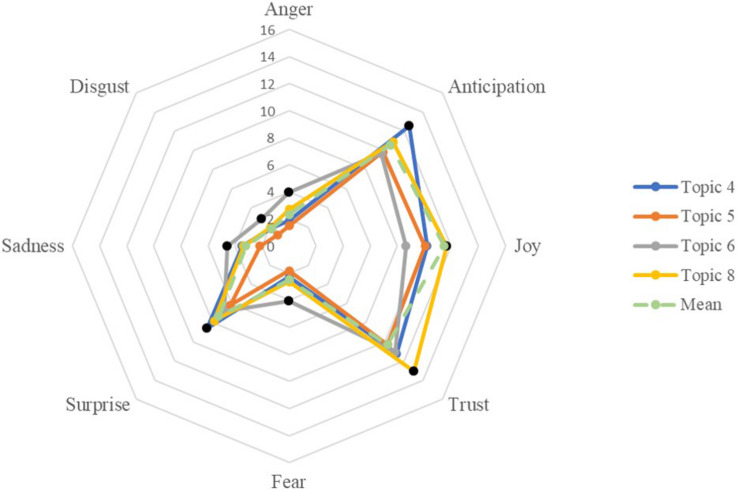
Composition of emotion of selected topics in Forum 1.

**FIGURE 5:**
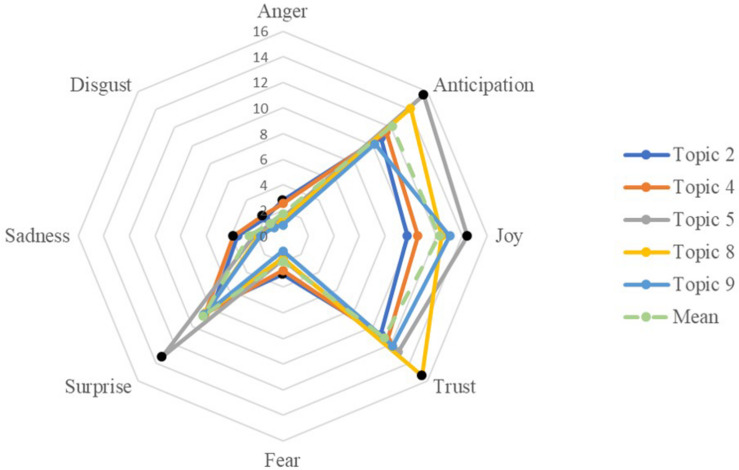
Composition of emotion of selected topics in Forum 2.

**FIGURE 6:**
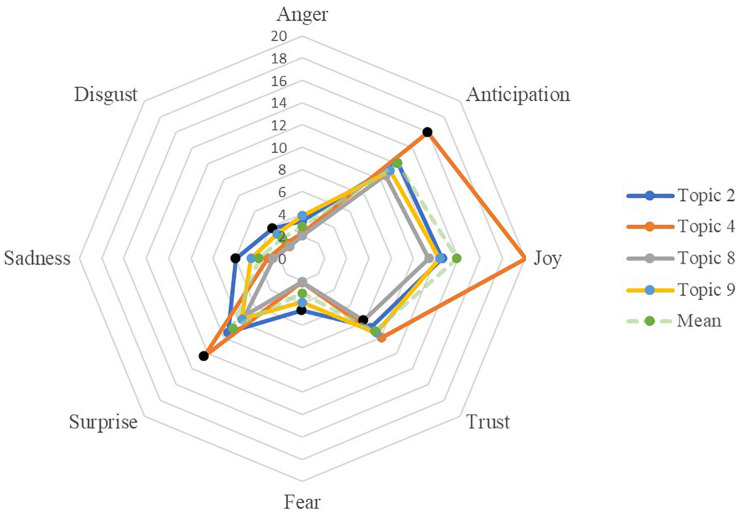
Composition of emotion of selected topics in Forum 3.

We further identified four representative topics in each forum which had the highest and lowest ratios of each emotion and presented them in three emotion radar charts in Figures 4–6. The radar charts mimicked the wheel structure of the eight emotions of Plutchik (1994) and easily demonstrated the weighted ratios of each emotion relative to others. In forum 1, as shown in Figure 4, topic 4 of *work schedule and commute* had the highest ratio in anticipation and surprise. Its other emotions, the ratios were close to the average forum values. Topic 8 (*ethnicity, gender, and religious diversity*) was the most emotional-rich topic in forum 1. It had the highest ratios of positive emotions in joy and trust and also high in anticipation and surprise. In terms of negative emotions, topic 6 of *conflicts in classrooms* had the highest ratios of multiple negative emotions, including anger, disgust, sadness, and fear. It also had the lowest ratio on joy. However, noticeably, comparing within topic 6, the ratio of joy is higher than the negative emotions. Topic 5 of *technologies in schools and teaching* tended to be less emotionally extensive. Its ratios of all emotion types were lower than the average numbers.

Similar to forum 1, the topics in forum 2 contained much more positive emotions than negative ones. In forum 2, topics 2 and 4, *behavior management* and *teaching pressures and health concerns*, respectively, had the highest ratios in the negative emotions in general. Moreover, their positive emotion ratios were lower than the forum-level averages, although their positive emotion ratios were still much larger than their negative ones. In contrast, topics 5, 8, and 9 contained the most positive emotions. Teachers shared the most anticipation, joy, and surprise in topic 5 of *interview tips and dress code*. When teachers are *seeking for job market advice*, as shown in topic 8, their discourses had the most trust and highest anticipation. Topic 9, *job mobility and relocation*, had a considerable amount of joy and the least of negative emotions compared with the other topics in this forum.

#### Emotions in Personal Life Events

Forum 3, which had the highest percentage of emotion words among forums, also was the most pronounced in showing variations of emotion ratio distributions and sentiment polarities across topics. In forum 3, topic 8 (*technology products*) is the least emotional-rich topic. This is similar to topic 5 of forum 1 which had a similar context. Forum 3’s topic 4 (i.e., *family and holidays*) is the most emotion-rich one. Specifically, topic 4 had the highest ratios of positive emotions and the smallest of negative emotions. In this topic, joy is the dominant emotion, which is 10 times larger than its sadness ratio. Topic 2 of *health concerns* and topic 9 of *beliefs of life and family* were highest in negative emotions. Topic 2 had ratio peaks in sadness, disgust, and fear, and topic 9 had a ratio peak in anger. In the sentiment boxplot, the median sentiment polarity scores were all positive. Identical to the patterns of emotion ratios, topics 2 and 9 are the lowest in terms of the positive sentiment scores, while topic 4 is the highest.

As the sentiment polarity of topics demonstrates similar patterns as the compositions of emotions did, we included sentiment polarity boxplots of topics in Supplementary Appendix B. First, all topics tend to be positive in the aggregated topic level sentiment since the thread-level polarity median estimates of all 30 topics were above zero. The ratios of emotion compositions and sentiment polarity medians showed consistent patterns at topic level within forums. For example, topic 5 in forum 1 is the most neutral as its forums’ sentiment polarity values were the closest to the zero points. Second, across forums, forum 2’s topics have a higher positive sentiment polarity in general. Forum 3 had the most pronounced variations of median sentiment polarity estimates across topics, while forum 1 had the least.

### Emotions Over a Decade

**FIGURE 7:**
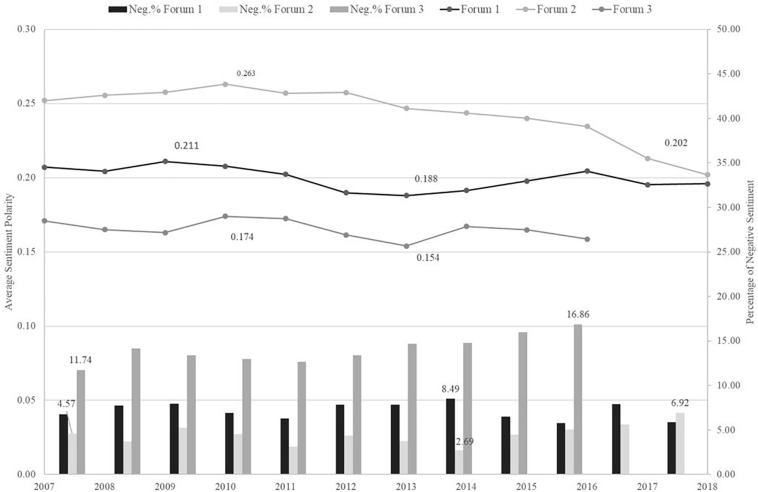
Trending plot of Average sentiment polarity and Negative sentiment percentage across Years.

**FIGURE 8:**
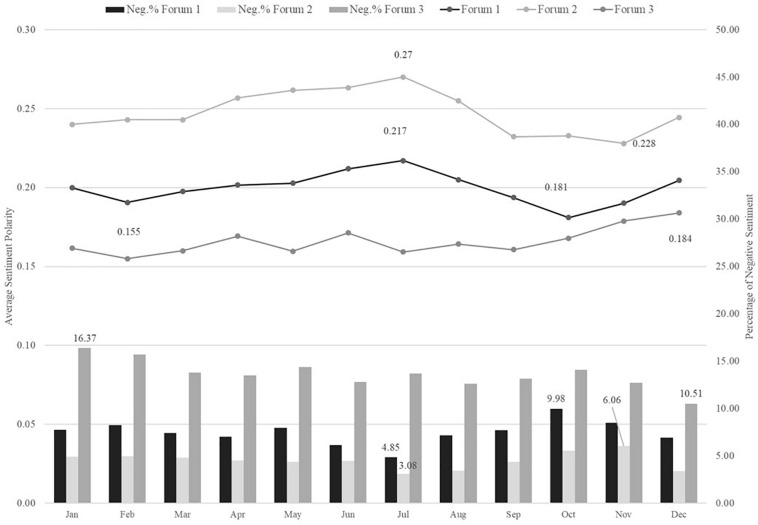
Trending plot of Average sentiment polarity and Negative sentiment percentage across Months.

**FIGURE 9:**
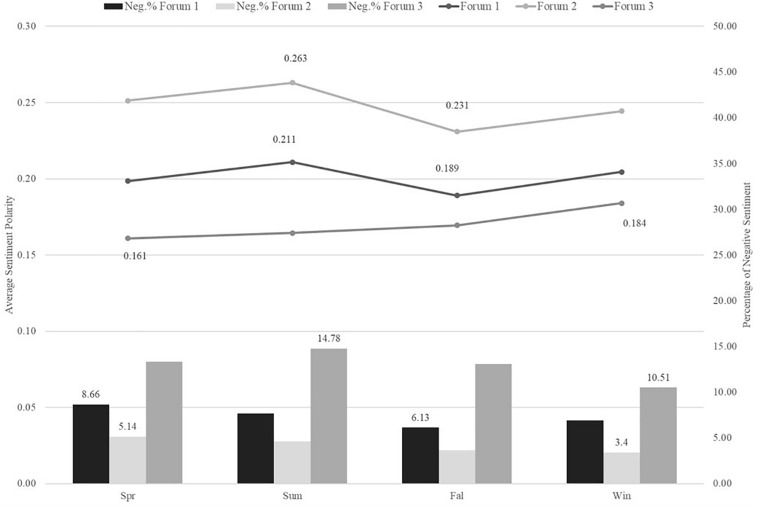
Trending plot of Average sentiment polarity and Negative sentiment percentage across Semesters.

In the trending plots (i.e., Figures 7–9) picturing average sentiment polarity estimates trajectories, we observed positive sentiments across all time measures. This is consistent with the above evidence of prevalent positive emotions. Forum 2 had the highest sentiment polarity across years, months, and semesters. In general, the average sentiment polarity scores were significantly different across the years, months, and semester terms of all three forums. All *p*-values were smaller than 0.01, except the test on months of forum 3.

In Figure 7 of the trending plot of years, forum 2 had a noticeable decline of average positive sentiments after 2013 compared with the other forums. Meanwhile, the percentage of negative sentiment threads in forum 2 increased from the lowest of 2.69% in 2014 to the highest of 6.92% in 2018. Forums 1 and 3 showed fluctuations instead of a monotonic trend. In 2013, both forums 1 and 3 had the lowest positive sentiment estimates of 0.19 and 0.15, respectively. Their sentiment polarity estimates’ maximum absolute changes between any 2 years were only one-third of the one in forum 2. These variations in change applied to time measures of months and semesters.

Across months shown in Figure 8, there were seasonality patterns that forums had increasing trends of positive sentiment from October to December and from April to July (besides forum 3). Similar patterns were found in the time measure of academic terms in Figure 9. In the semesters of spring and fall, there were slumps, while in the breaktime of summer and winter the average positive sentiment estimates hit peaks. These seasonality fluctuations were less reflected in forum 3 though. It had a general increasing trend across months and semesters and hit the peak in winter times.

The two-way ANOVA tests also detected significant interaction effects of topics and time in forums 1 and 2 (all *p*-values are smaller than 0.01). Forum 3 only had a significant interaction effect of topics and months (*p* = 0.005). We then dived into prevalent topics that had significant changes in their sentiment scores across time matrices. In forum 2, topic 8 of *job market advice-seeking* had a noticeable and stable sentiment polarity decreasing trend as the years pass. The decline of the positive sentiment polarity degrees happened along with the decrease in the weighted ratios of positive discrete emotions. The ratio statistics of joy and anticipation in this topic decreased by 20% from 2007 to 2018. The emotion of surprise even decreased by 40% in ratios. The ratios of the other emotions were relatively stable, including trust.

Another exemplar of topic 2, *behavior management*, had ratios of negative emotions of anger and disgust that increased to around 40%, and fear and sadness increased to around 20% from summer to fall semester. The corresponding transitional months were July and September. Meanwhile, the positive emotions’ ratios decreased in a range of 10–20%. Another classroom behavior management topic (i.e., topic 9 of forum 1) had a similar increasing trend in negative emotions during summer to fall. The increase rates in this topic were 10–30%. Although no significant interaction effect of topic and semester was found in forum 3, its topic 4, *family and holidays*, is a good example to show the increasingly positive sentiment in winter. From fall to winter semesters, its weighted ratios of positive emotions increased by around 10–25%, and anger and disgust decreased by around 40 and 20%, respectively. The other negative emotions remained low.

## Discussion and Conclusion

This paper utilized a longitudinal text dataset with emotion-rich information from teachers’ online professional forums to reveal the complexity of teacher emotions. By computational text analysis tools, traditional statistical analysis (i.e., ANOVA), and extensive visualizations, we provided a variety of empirical evidence. Although driven by the emergent big data and the computational text analysis tools in a mainly exploratory approach, the research goals and designs were driven by and weighed heavily on the contemporary theories from teacher emotion studies. We discussed the patterns of teachers’ emotions and sentiments in forums and extended to the many specific workplaces and personal life contexts and three time measures. Additionally, the online text dataset with a large sample of American teachers and an extensive time range of 10 years enlarged the external validity of this paper. In the following discussion, we summarize the findings to support the current theoretical argument of the complexity of emotions: multi-dimensionality and dynamic. We also discussed novel perspectives for future studies.

### Multi-Dimensionality of Teacher Emotions

Embedded within the many educational contexts (i.e., topics) computed from teachers’ online professional forums, teachers exhibit a composite of multiple types of emotions. This empirical evidence extended the multi-dimensionality character of emotions as defined in Weiss and Cropanzano (1996) and Fredrickson (2001) of large teacher groups.

Our first research question was answered. In general, all eight discrete emotions defined by Plutchik (1994) were detected in teachers’ online discourses and discussions through rule-based text analyses at the forum, topic, and time level. In other words, teachers have varying emotions simultaneously, ranging from positive to negative.

In certain educational contexts that were negatively valued, only the negative emotions of teachers may have caught the attention of researchers. However, the current paper’s findings suggested that positive emotions exist simultaneously. Sutton and Wheatley (2003) have made the same critiques for the traditional survey studies where negative-valued events may lead researchers to less likely track positive emotions. Also, these positive emotions were easily diluted if teachers’ emotional manifestations were measured by the overall and the aggregated negative valence. In contrast, the computational sentiment analysis can efficiently provide a comprehensive list of discrete types of emotion estimates to uncover the hidden or less displayed positive emotions.

An example is topic 6 (*conflicts in classrooms*) of forum 1, which is a commonly negative-valued context. In this topic, additional to the negative emotions, a considerable portion of joyful emotion concurrently exists. Similar patterns showed in the two behavior management topics from forums 1 and 2. They are consistent with the prior research findings that student discipline and teacher–student relationship are significant factors of teachers’ positive (such as joy) and negative (such as anxiety and anger) emotions (Hagenauer et al., 2015). There was evidence showing that teachers who adopted adaptive coping and emotion regulation strategies would ease their unpleasant emotions (Chang, 2009, 2013; Cross and Hong, 2012; Yin, 2016) or teachers would experience positive emotions when their students made improvements of behavior management in classes, such as more engagement in learning (Hagenauer et al., 2015; de Ruiter et al., 2019). It is possible that teachers may have an increased likelihood of having positive emotions due to their accumulated experiences in dealing with classroom and student behavior management issues (Golby, 1996; Sutton, 2004). Therefore, when teachers encountered similar issues, their appraisal process may be different from their previous experience and then they may show emotional compositions differently than before.

A few studies have shown that there were potential neglected effects of positive or negative emotions in the opposite valence-valued contexts. In Hagenauer et al. (2015), they found that teachers showed emergent joy when a close teacher–student relationship exists and, further, that the close-exchange relationship would lead to less frequent anxiety and anger. Without measuring and testing a comprehensive list of emotions of teachers that could potentially play interactively and compensate for each other, the influence of the emotions on teachers and students may be biased. In the results, although sentiments all tended to be positive, negative discrete emotions actually exist. If only polarities were used for inferential analysis, the overlooked negative emotions could play some hidden impacts of suppressing the effects of positive emotions.

Another significant feature of teachers’ emotional multi-dimensionality shown in the results is the pronounced positive emotions and sentiments. Although the eight emotions of positive and negative ones commonly appear simultaneously, teachers tend to express more positive emotions than negative ones across all topics. This is also true even in the contexts that are normally negatively perceived, such as the topic of students’ behavior management. This pattern is consistent with many diary studies of recording teachers’ discrete emotions in their teaching sessions and workdays (Carson, 2007; Becker, 2011), as summarized in Frenzel (2014). In these studies, enjoyment was the most noticeable positive emotion, which accounts for more than 70% of the overall emotions. Anger, correspondingly, is the primary negative emotion, but its frequency is four times less than enjoyment. Also, teachers, particularly female teachers, often suppress their negative emotional expressions due to their workplace power structures (Liljestrom et al., 2007) or social norms (Zhu et al., 2014). This feature also suggests that the simple binary classification of emotion and sentiment valence (i.e., positive or negative) would limit disclosing of the complexity of teachers’ emotions. In general, teachers’ average positive sentiment polarity degrees were pronounced, while negative emotions accounted for non-trivial proportions.

### Dynamics of Teacher Emotions

This study further explored the dynamics of emotions as the other characteristic of the complexity of teachers’ emotions. It argues the variations and changing status of emotions and degrees of sentiment polarity across contexts and time. Embedded within particular topics, evidence showed certain emotions or emotion combinations dominate. This finding made contributions to the current teachers’ emotions with a more diverse range of contexts. As Frenzel (2014) noted, current knowledge mainly narrowed within the context of classroom teaching activities. Previous studies gathered much knowledge in examining teachers’ emotions in the contexts of teachers–students relations and students’ behavior management in classrooms (Greene et al., 1997; Chang and Davis, 2009; Spilt et al., 2011; McGrath and Van Bergen, 2017; Koenen et al., 2019). However, there are less scholarly studied educational contexts that are critical to teachers’ emotions and professions that need attention.

For example, this paper provided many topics that were relevant to teachers’ corporations outside of local schools, such as topics 1, 5, 8, 9, and 10 in forum 2 about sharing and seeking suggestions for jobs and interviews. In these topics, we observed considerable ratios of trust, along with high ratios of other positive emotions, compared with other topics. This implies to some empirical studies finding that trust among teachers and their communities increase teachers’ knowledge sharing (Bulu and Yildirim, 2008; Chen et al., 2014) with the compliance of enjoyment (Kankanhalli et al., 2005). However, few teacher emotion studies have explored the context of teacher’s community and collaboration and whether the occupied trust and other positive emotions would ease teachers’ negative emotions in their classroom teaching.

Though as shown in the aforementioned literature that teachers’ personal events could influence teachers’ school activities and professional experience, current literature still knows little about how teachers’ personal life events may affect teachers’ emotions. The current study is the first to show teachers’ emotions in their out-of-school life through two major evidence. Firstly, teachers expressed emotional rich discourses that situated within a wide range of outside-of-school topics, such as family life and holiday, health concerns, and leisure time entertainment. Secondly, the current paper also further found noticeable differences of teachers’ emotional manifestos between school-based activities and personal life events. Specifically, teachers tended to express more negative emotions and sentiments in their personal life related topics than in workspace events. These outside-of-school events are rarely considered in teachers’ emotions studies while are crucial components in individual teachers’ daily life and influence teachers’ school-based activities and emotions. For example, teachers who have conflicts in work and family may less likely to have positive engagements and experience in school activities (Bragger et al., 2005).

The other feature of the dynamics of teacher emotions is time. This feature is less empirically studied mainly due to the limited capacity of survey or interview data with traditional qualitative or quantitative inquires (Frenzel, 2014). Benefited from big data’s extensive time range of 10 years and the computational ability of text analysis tools, this study found significant changes in emotion compositions and average positive sentiment degrees across months, years, and academic semesters. The dynamics of teacher emotions also evidenced through the interaction effects of time and topics. In the example topics discussed, we found increased negative emotions during academic semesters in several workplace topics, while increased positive emotions in winter breaks of personal life event. This evidence nicely reflected the seasonality of teaching professions and the corresponding teachers’ emotional experience and changes.

## Limitations and Future Studies

Leveraging teachers’ online text data with computational text analysis methods, this paper presented an overall picture of the complexity of teachers’ emotions in a large scale and in an efficient way. However, we did not provide micro-level examinations of the many appraisal processes of particular emotions at the individual teacher level. The appraisal process is complicated as it involves an entire ecosystem of teachers’ own identity and psychological status, students’ behaviors, school dynamics, and the broader educational policy system (Bronfenbrenner and Morris, 1998; Cross and Hong, 2012). This requires us to combine qualitative analysis for in-depth evidence of individual teachers’ appraisals within narrower contexts to forward this paper, which covered wide ranges through computational text analysis. For example, we need to understand the appraisal process involved in the event that teachers express positive emotions in the negatively valued events, such as dealing with students’ improper behaviors, during an adequately longer timeframe. Furthermore, what were the characteristics and the actual classroom experience that these teachers had? We also provided evidence that teachers’ sentiment tends to be positive on average across forums, topics, and time. In contrast, compared with discrete emotion compositions, many emotion types on the opposite sentiment exist. However, we still know little whether the positive and the negative emotions have an interactive effect that may either reinforce or weaken each other and influence teachers’ appraisal process. These studies can further enrich our understanding of teacher emotions. For example, this knowledge can significantly help teacher preparation and development programs to facilitate teachers’, particularly novice teachers’, emotional competency and capital improvements (Cross and Hong, 2012).

This paper also demonstrated the affordances of big data and computational text analysis in teachers’ emotions studies. We provided discrete emotions’ compositions and sentiment polarity estimates, which advanced the current measures of teachers’ emotions. These measures obtained from teachers’ online text captured less subjectively biased emotion measures than the traditional survey or interview approach. However, teachers may hide or fake their genuine emotions (Taxer and Frenzel, 2015). The source of joy and other positive emotions, as shown in this paper, may be generated by the norms of the online forums, where people were found to more likely express positive sentiments and emotions in online forums and social media sites (Waterloo et al., 2018). As females could account for around 90% of the teacher population in American’s elementary schools (McFarland et al., 2019), the positive sentiment and emotion manifesto could be also be impacted by potential gender effects. However, the current study has no information on users’ gender, which is commonly seen as a shortcoming of big data scraped from public online platforms. We therefore encourage future studies to conduct interviews and surveys to examine whether these responding biases exist in teachers’ online data and also compare with data collected by the traditional interview or survey means.

## Data Availability Statement

Publicly available datasets were analyzed in this study. This data can be found here: http://atozteacherstuff.com/.

## Author Contributions

ZC generated the original ideas, led in designing the study, conducted the analyses, wrote the first draft on introduction, methods, results, and discussion. XS developed the original ideas, helped in research design and analyses, wrote the first draft on introduction and literature review, gave feedback, and helped ZC to edit the final draft of the manuscript. LQ and WZ contributed to idea generation, draft finalization, and journal submission.

## Conflict of Interest

The authors declare that the research was conducted in the absence of any commercial or financial relationships that could be construed as a potential conflict of interest.
