# Supplementary material for: Understanding the Complexity of Teacher Emotions From Online Forums: A Computational Text Analysis Approach
Source: Front Psychol. 2020 Jun 5;11:921. doi: 10.3389/fpsyg.2020.00921 (PMC7290013; doi:10.3389/fpsyg.2020.00921)

**Appendix A**

Weighted Ratios of Emotions by Topics of Forums

| Topics in Forum 1  (Prevalence %) | | Anger | | Sad-ness | | Dis-gust | | Fear | | Surp-rise | | Antic-pation | | Joy | | Trust |
| --- | --- | --- | --- | --- | --- | --- | --- | --- | --- | --- | --- | --- | --- | --- | --- | --- |
| 1 | Professional training investment  (13.8) | 1.94 | | 2.89 | | 1.45 | | 2.04 | | 7.55 | | 11.31 | | 10.83 | | 12.90 |
| 2 | Teaching literacy & writing  (12.1) | 1.92 | | 2.37 | | 1.28 | | 1.98 | | 7.06 | | 10.46 | | 10.12 | | 11.38 |
| 3 | Teaching physical resources  (10.7) | 1.95 | | 2.58 | | 1.62 | | 1.91 | | 7.22 | | 10.17 | | 10.42 | | 9.86 |
| 4 | Work schedule & commute  (9.6) | 1.87 | | 3.49 | | 1.85 | | 2.29 | | 8.58 | | 12.54 | | 10.20 | | 11.22 |
| 5 | Technologies in schools & teaching  (11.6) | 1.51 | | 2.16 | | 1.16 | | 1.82 | | 6.16 | | 9.82 | | 10.06 | | 10.31 |
| 6 | Conflicts in classrooms  (7.4) | 3.96 | | 4.54 | | 2.86 | | 4.07 | | 6.81 | | 9.65 | | 8.65 | | 11.08 |
| 7 | Testing & special ed. Evaluation  (9.0) | 2.06 | | 3.09 | | 1.46 | | 2.48 | | 6.51 | | 9.56 | | 9.43 | | 11.73 |
| 8 | Ethnicity, gender, & religious diversity  (8.0) | 2.70 | | 3.38 | | 1.93 | | 2.63 | | 7.81 | | 10.88 | | 11.66 | | 13.08 |
| 9 | Behavior management  (10.5) | 2.60 | | 3.51 | | 2.03 | | 2.71 | | 7.70 | | 10.59 | | 10.35 | | 11.27 |
| 10 | Teacher dressing code  (7.3) | 2.47 | | 3.36 | | 2.43 | | 2.58 | | 8.36 | | 10.78 | | 12.36 | | 10.63 |
|  | Forum-level average | 2.34 | | 3.20 | | 1.81 | | 2.50 | | 7.37 | | 10.58 | | 11.46 | | 10.31 |
| Topics in Forum 2  (Prevalence %) | | | Anger | | Sad-ness | Dis-  gust | Fear | | Surp-rise | | Antic-  ipation | | Joy | | Trust | |
| 1 | Job offer choices  (12.5) | | 1.65 | | 2.70 | 1.36 | 1.79 | | 9.41 | | 13.00 | | 11.51 | | 11.65 | |
| 2 | Behavior management  (8.2) | | 2.79 | | 3.54 | 1.97 | 3.02 | | 7.68 | | 10.88 | | 9.74 | | 10.87 | |
| 3 | Educational resource and supplies  (10.0) | | 1.63 | | 2.28 | 1.16 | 1.71 | | 8.28 | | 12.14 | | 11.31 | | 11.42 | |
| 4 | Teaching pressure and health concerns  (8.8) | | 2.55 | | 3.84 | 2.25 | 2.68 | | 8.63 | | 11.45 | | 10.55 | | 11.58 | |
| 5 | Interview tips and dress code  (10.0) | | 1.45 | | 2.30 | 1.35 | 1.84 | | 13.36 | | 15.56 | | 14.47 | | 12.78 | |
| 6 | Professional training and certification  (10.1) | | 1.39 | | 2.16 | 1.08 | 1.66 | | 8.07 | | 10.81 | | 10.84 | | 12.36 | |
| 7 | General teaching practice and student learning  (9.6) | | 1.59 | | 2.59 | 0.98 | 1.86 | | 6.74 | | 9.83 | | 10.28 | | 12.15 | |
| 8 | Job market advice seeking  (12.0) | | 1.28 | | 1.91 | 1.03 | 1.73 | | 8.53 | | 14.13 | | 12.44 | | 15.42 | |
| 9 | Job mobility and relocation  (4.5) | | 0.87 | | 1.77 | 0.94 | 1.18 | | 8.70 | | 10.19 | | 13.07 | | 12.15 | |
| 10 | Job application systems and procedures  (14.3) | | 1.28 | | 2.16 | 1.32 | 1.54 | | 9.06 | | 13.26 | | 9.77 | | 11.28 | |
|  | Forum-level average | | 1.74 | | 2.64 | 1.41 | 1.98 | | 8.81 | | 12.14 | | 12.23 | | 11.23 | |

| Topics of Forum 3  (Prevalence %) | | Anger | Sad-ness | Dis-  gust | Fear | Surp-rise | Antici-pation | Joy | Trust |
| --- | --- | --- | --- | --- | --- | --- | --- | --- | --- |
| 1 | Bills and investment  (6.6) | 3.36 | 3.46 | 1.91 | 2.68 | 8.81 | 12.06 | 12.56 | 9.77 |
| 2 | Health concerns  (11.1) | 3.36 | 5.92 | 3.80 | 4.65 | 9.38 | 12.13 | 12.59 | 8.73 |
| 3 | Leisure time entertainment  (12.4) | 3.22 | 4.15 | 2.85 | 3.49 | 9.63 | 12.93 | 15.88 | 9.55 |
| 4 | Family and holidays  (10.3) | 2.25 | 3.09 | 1.97 | 2.12 | 12.44 | 16.01 | 20.09 | 10.04 |
| 5 | Meal and cooking  (7.7) | 2.30 | 2.85 | 2.39 | 2.57 | 8.88 | 11.89 | 16.70 | 8.92 |
| 6 | House and classroom organization  (11.1) | 2.34 | 3.56 | 2.72 | 2.36 | 7.64 | 10.27 | 12.97 | 8.45 |
| 7 | Education concerns and professional growth  (9.6) | 2.29 | 3.18 | 1.79 | 2.50 | 8.22 | 12.36 | 12.03 | 11.01 |
| 8 | Technology products  (10.9) | 2.02 | 2.61 | 1.54 | 2.08 | 7.24 | 10.58 | 11.39 | 7.82 |
| 9 | Beliefs of life and family  (10.3) | 3.83 | 4.59 | 3.16 | 3.92 | 7.65 | 11.15 | 12.35 | 9.41 |
| 10 | Vacation and travel  (10.1) | 2.79 | 3.38 | 2.08 | 3.03 | 8.83 | 12.29 | 13.78 | 8.03 |
|  | Forum level average | 2.87 | 3.87 | 2.56 | 3.10 | 8.82 | 12.12 | 13.88 | 9.22 |

**Appendix B**

Boxplots of Sentiment Polarity Estimates across Topics in Forums
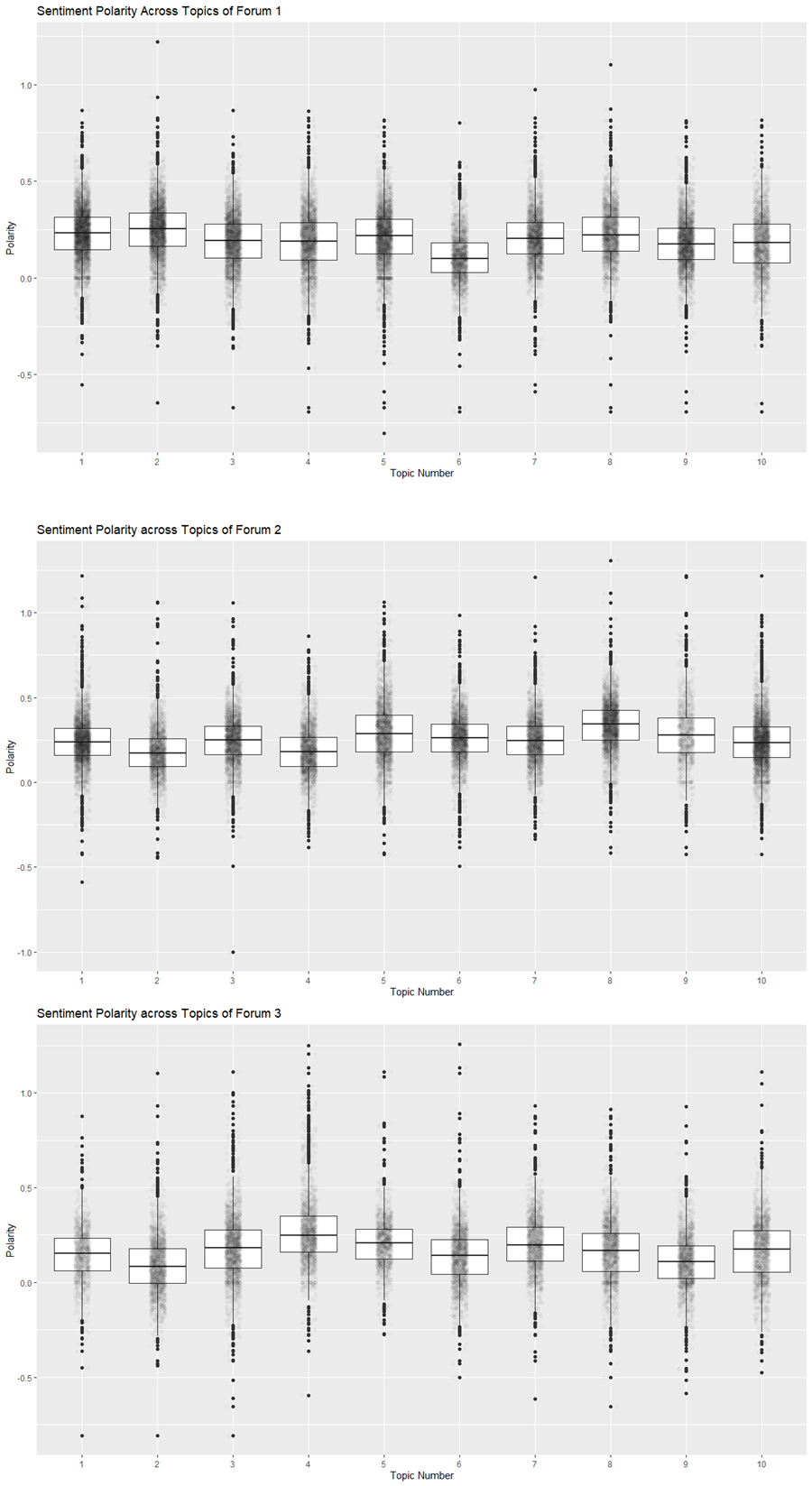


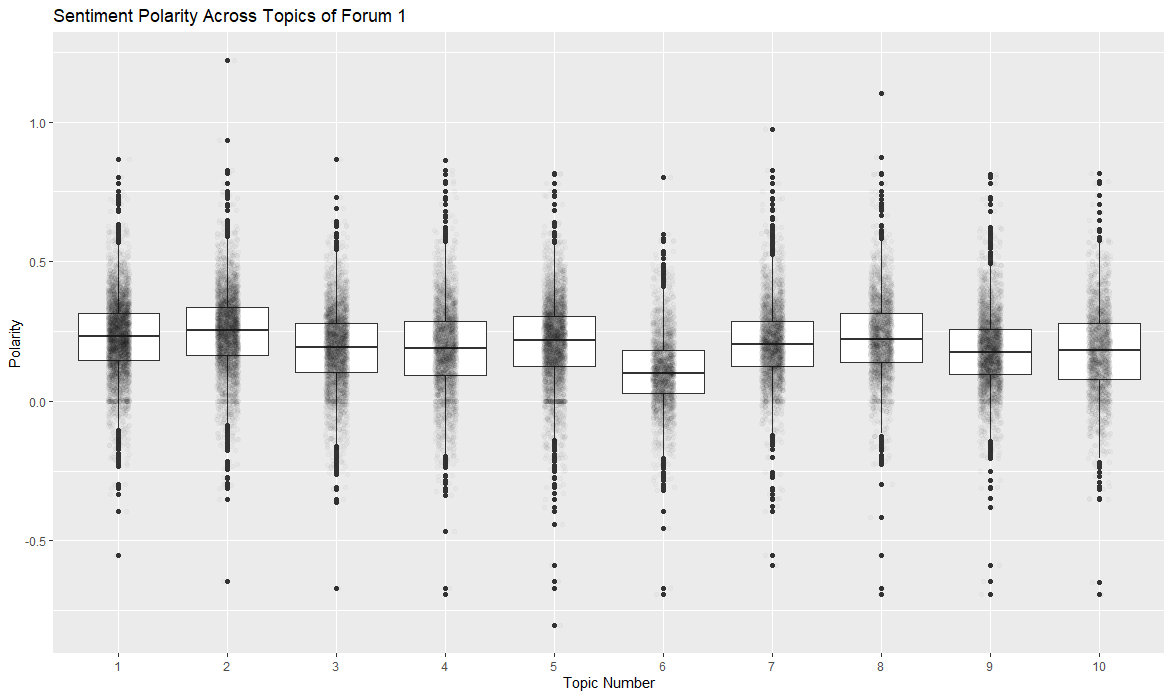


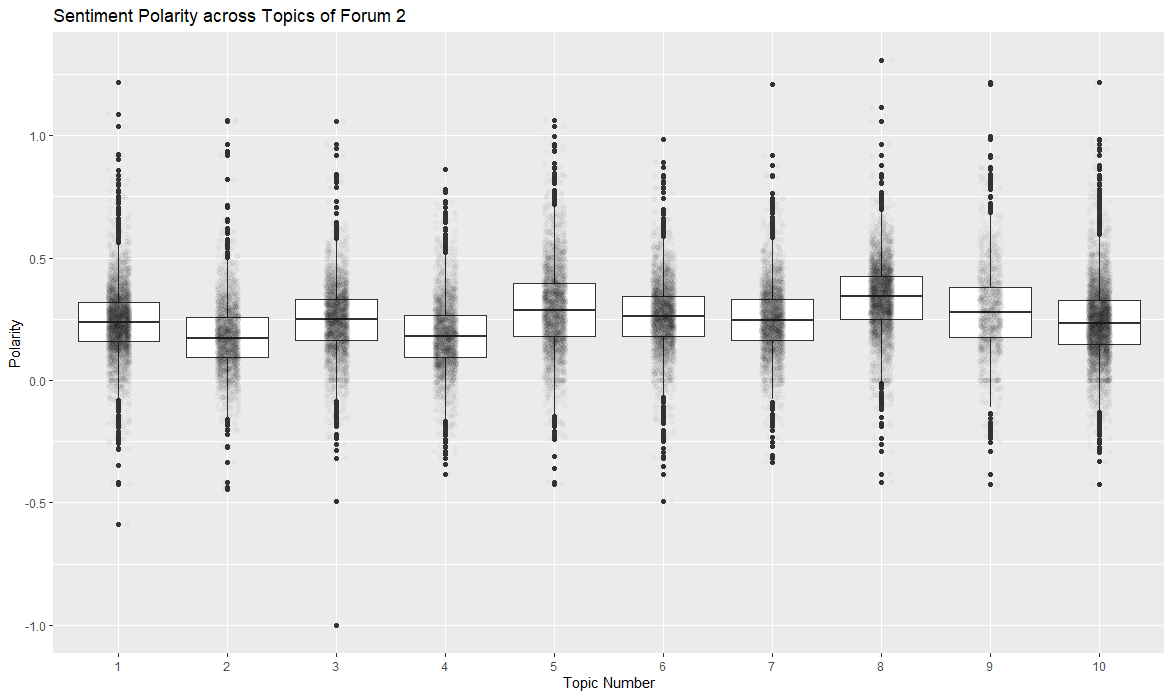


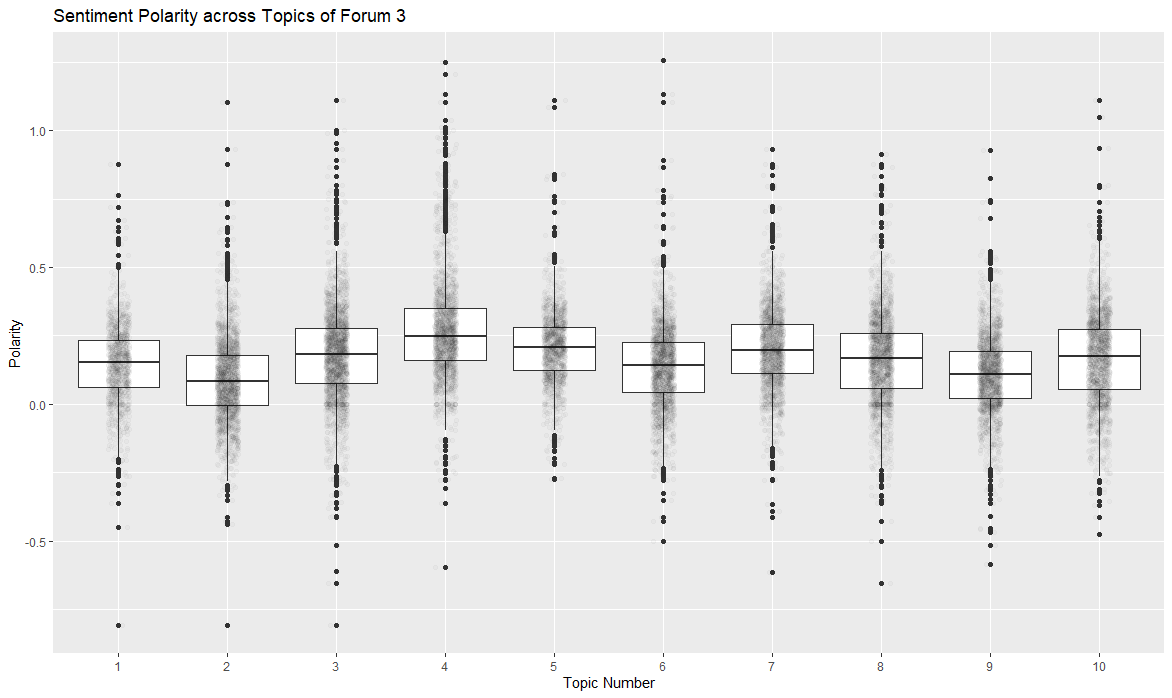

Supplement: Supplementary file 1 [file Table_1.DOCX]
